# Leptospirosis-Associated Hospitalizations, United States, 1998–2009

**DOI:** 10.3201/eid2008.130450

**Published:** 2014-08

**Authors:** Rita M. Traxler, Laura S. Callinan, Robert C. Holman, Claudia Steiner, Marta A. Guerra

**Affiliations:** Centers for Disease Control and Prevention, Atlanta, Georgia, USA (R.M. Traxler, L.S. Callinan, R.C. Holman, M.A. Guerra);; Agency for Healthcare Research and Quality, Rockville, Maryland, USA (C. Steiner)

**Keywords:** leptospirosis, Leptospira, hospitalization, adults, humans, seasonality, United States, epidemiology, environmental exposure, hospitalization statistics and numerical data, length of stay, re-emerging, reemerging, zoonoses, bacteria, incidence

## Abstract

A small percentage of persons with leptospirosis, a reemerging zoonosis, experience severe complications that require hospitalization. The number of leptospirosis cases in the United States is unknown. Thus, to estimate the hospitalization rate for this disease, we analyzed US hospital discharge records for 1998–2009 for the total US population by using the Nationwide Inpatient Sample. During that time, the average annual rate of leptospirosis-associated hospitalizations was 0.6 hospitalizations/1,000,000 population. Leptospirosis-associated hospitalization rates were higher for persons >20 years of age and for male patients. For leptospirosis-associated hospitalizations, the average age of patients at admission was lower, the average length of stay for patients was longer, and hospital charges were higher than those for nonleptospirosis infectious disease–associated hospitalizations. Educating clinicians on the signs and symptoms of leptospirosis may result in earlier diagnosis and treatment and, thereby, reduced disease severity and hospitalization costs.

## Abstract 2

Average cost and duration of hospitalizations were significantly greater than for other infectious diseases.

Leptospirosis is a bacterial zoonotic infection caused by pathogenic serovars in the genus *Leptospira* (*1*). Approximately 10% of infections in humans result in clinical disease characterized by abrupt onset of fever, headache, muscle aches, and gastrointestinal involvement (*2*,*3*). Some infected persons can experience biphasic illness, in which more severe symptoms begin after a short recovery period (*2*,*3*). A total of 10%–15% of patients with clinical disease experience severe leptospirosis, characterized by multiple organ involvement (e.g., renal and liver failure, pulmonary distress and hemorrhage, cardiac arrhythmia), and a high rate of death (*2*,*3*). Severe infections comprise the majority of reported cases, but these cases underrepresent the incidence of disease (*4*).

Leptospirosis has historically occurred in persons who have contact with fresh water following heavy rains and in persons who work outdoors, with animals, or in wet environments contaminated with animal urine (*2*,*3*,*5*). The disease occurs more frequently in adult men than in children or women (*4*,*6*), and it is most prominent during warm and rainy seasons (*2*,*3*). In the United States, new groups at risk for leptospirosis have emerged, including residents in urban areas (*7*) and participants in freshwater sports (*8*,*9*).

In most places worldwide, leptospirosis is considered a reemerging human and animal disease (*1*,*5*). However, the disease was not considered nationally notifiable during 1995–2012, so whether human leptospirosis is reemerging in the United States is unknown (*10*). During those years, leptospirosis was reportable in many states; among them, California and Hawaii showed reemergence of the disease (*11*,*12*). In addition, a report describing a higher than expected death rate among leptospirosis-infected persons in Puerto Rico suggested that, on the basis of the average death rate, many more clinical cases of leptospirosis should have been reported (*13*). The fewer than expected number of reported cases might have resulted from underreporting or from a lack of disease recognition. The findings in those reports indicate the potential reemergence of leptospirosis as a public health problem in the United States.

To increase our knowledge of this neglected disease in the United States, we used national hospital discharge data for 1998–2009 to estimate the number of persons in the US population with symptomatic leptospirosis requiring hospitalization. We also used the discharge data to evaluate trends of leptospirosis-associated hospitalizations during the study period and to compare hospitalizations for leptospirosis with those for other infectious diseases.

## Methods

We analyzed the general US population hospital discharge data for 1998–2009 from the Nationwide Inpatient Sample (NIS) (*14*). The Healthcare Cost and Utilization Project (HCUP), sponsored by the Agency for Healthcare Research and Quality (Rockville, MD, USA), produces NIS in collaboration with participating states (*15*). NIS is the largest all-payer inpatient care database in the United States and is a nationally representative sample of hospitals that includes a 20% sample of participating US community hospitals. Participating hospitals are short-term, nonfederal general and specialty hospitals sampled annually from up to 44 states. The overall design objective of NIS is to select a sample of hospitals that accurately represents the US population (*15*).

We calculated national estimates of the number of hospitalizations in the United States by using the HCUP weighting method (*15*,*16*). SEs and 95% CIs for rates were calculated by using SUDAAN software (http://www.rti.org/sudaan/). If the relative SE (i.e., SE/no. of estimated hospitalizations) of an estimate was >0.30 or if unweighted counts were <10.0, data were suppressed because the estimate was considered unreliable (*15*,*16*). The unit of analysis was a hospitalization; birth-associated hospitalizations were excluded from the analysis.

For analysis, we selected hospitalizations during 1998–2009 with an International Classification of Diseases, 9th revision, Clinical Modification code (ICD-9-CM code) for leptospirosis (i.e., code100) listed as any 1 of up to 15 diagnoses on the hospitalization record (*17*). We calculated annual and average annual leptospirosis-associated hospitalization rates (per 1,000,000 persons) for the study period by using the annual number of weighted leptospirosis-associated hospitalizations and the corresponding annual census population overall and by sex, age group, and census region. Denominators were estimated by using the annual bridged race population estimates for 1998–2009 from the National Center for Health Statistics, US Centers for Disease Control and Prevention (*18*,*19*). Regions, defined by HCUP, were based on the US census regions (Northeast, South, Midwest, and West) (*20*), which do not include US territories. Leptospirosis-associated hospitalizations were compared with nonleptospirosis infectious disease–associated hospitalizations, which were defined as hospitalizations for a first-listed infectious disease, as defined in previous studies (*21*) with updates as appropriate, other than leptospirosis. We calculated rate ratios to compare rates between groups (*22*,*23*). Hospitalizations were not examined by patients’ race/ethnicity because these data were missing in 19% of the records.

For patients with leptospirosis-associated hospitalizations, we calculated the mean and median age at admission overall and by sex. We examined seasonality for leptospirosis-associated hospitalizations by month of patient admission during the study period. We calculated the mean and median hospital charges for leptospirosis-associated hospitalizations overall, and we calculated the mean and median length of stay by the age and sex of patients and by region. Age, hospital charges, and length of stay for leptospirosis-associated hospitalizations were also compared with those for nonleptospirosis infectious disease–associated hospitalizations. We performed *t*-tests in SUDAAN to determine whether leptospirosis-associated hospitalization charges and lengths of stay differed significantly by sex and region (*24*).

## Results

**Figure 1:**
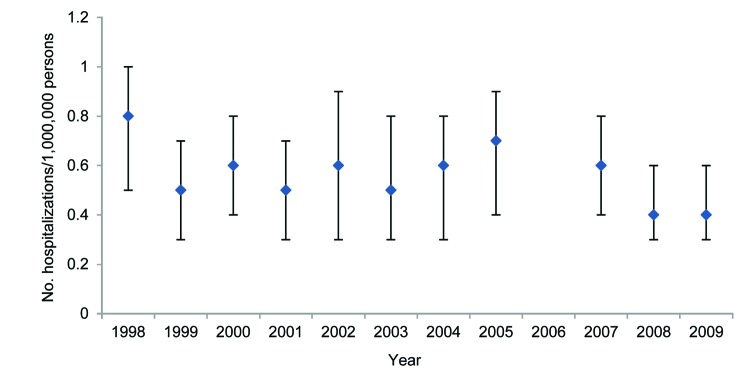
Yearly rate of leptospirosis-associated hospitalizations, United States, 1998–2009. Vertical bars indicate 95% CIs. The rate for 2006 is not included because it was unstable (relative SE >0.3).

During 1998–2009 in the United States, the average annual rate of leptospirosis-associated hospitalizations was 0.6 hospitalizations/1,000,000 population (95% CI 0.5–0.6) (Table 1); the annual rate did not change over the period (Figure 1). Regional average annual rates ranged from 0.4 hospitalizations/1,000,000 population (95% CI 0.3–0.5) in the Northeast to 0.7 hospitalizations/1,000,000 population (95% CI 0.5–0.9) in the West (Table 1).

**Table 1:** Leptospirosis-associated hospitalizations and hospitalization rates by selected demographic characteristics, United States, 1998–2009*

Characteristic	No. leptospirosis-associated hospitalizations (SE)†	Hospitalization rate (95% CI)‡
Total	1,994 (126)	0.6 (0.5–0.6)
Patient age group		
0–19	287 (41)	0.3 (0.2–0.4)
20–59	1,260 (95)	0.7 (0.6–0.7)
>60	441 (53)	0.7 (0.6–0.9)
Patient sex, age group, y		
M	1,401 (105)	0.8 (0.7–0.9)
0–19	190 (32)	0.4 (0.3–0.5)
20–59	934 (80)	1.0 (0.8–1.1)
>60	277 (42)	1.1 (0.8–1.4)
F	587 (57)	0.3 (0.3–0.4)
0–19	97 (22)	0.2 (0.1–0.3)
20–59	326 (42)	0.3 (0.3–0.4)
>60	164 (31)	0.5 (0.3–0.7)
Region of residence		
Northeast	261 (37)	0.4 (0.3–0.5)
Midwest	387 (48)	0.5 (0.4–0.6)
South	780 (68)	0.6 (0.5–0.7)
West	565 (87)	0.7 (0.5–0.9)

*SEs and 95% CIs were calculated by using SUDAAN software (http://www.rti.org/sudaan/). †Numbers in subgroups do not total 1,994 because of missing values in the Nationwide Inpatient Sample. ‡Rate per 1,000,000 persons in corresponding population.

The mean age of US patients with leptospirosis-associated hospitalizations was significantly younger than that for US patients with nonleptospirosis infectious disease–associated hospitalizations (43.2 y [SE 1.1] vs. 52.1 y [SE 0.2]; p<0.001) (median ages are shown in Table 2). The mean age of female patients with leptospirosis-associated hospitalizations was slightly older than that for male patients, but the difference was not statistically significant (45.7 y [SE 2.2] vs. 42.1 [SE 1.2]; p = 0.15).

**Table 2:** Numbers of leptospirosis-associated and nonleptospirosis infectious disease–associated hospitalizations by selected variables, United States, 1998–2009

Variable, characteristic	Median no. (25th, 75th quartiles) hospitalizations
Patient age, y	
Infection type	
Leptospirosis-associated	42.1 (27.8, 57.4)
Nonleptospirosis infectious disease	56.4 (30.4, 76.2)
Sex of patient	
M	40.8 (27.0, 55.4)
F	44.2 (30.2, 60.6)
Length of hospital stay, d, by hospitalization type	
Leptospirosis-associated	4.1 (2.4, 7.5)
Nonleptospirosis infectious disease–associated	3.3 (1.7, 6.0)
Hospital charges, US dollars, by leptospirosis-associated hospitalizations	
Total	19,768 (10,444, 37,422)
Sex of patient	
M	18,577 (11,161, 34,855)
F	24,093 (9,279, 44,960)
Patient age group, y	
<20	17,815 (9,253, 33,780)
20–59	18,942 (10,700, 35,046)
>60	24,578 (10,230, 58,103)

The leptospirosis-associated hospitalization rate for adults 20–59 years of age (0.7 hospitalizations/1,000,000 corresponding population, 95% CI 0.6–0.7) and >60 years of age (0.7 hospitalizations/1,000,000 corresponding population, 95% CI 0.6–0.9) differed from the rate for persons 0–19 years of age (0.3 hospitalizations/1,000,000 corresponding population, 95% CI 0.2–0.4) (Table 1). The leptospirosis-associated hospitalizations rate for male patients was 2.5 times the rate for female patients (95% CI 1.9–3.1, p<0.001).

**Figure 2:**
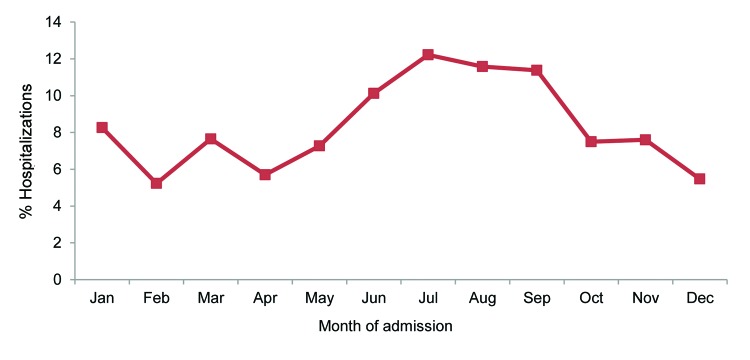
Monthly percentages of leptospirosis-associated hospitalizations, United States, 1998–2009.

A high proportion of leptospirosis-associated hospitalization admissions occurred during June–September (41.2% [SE 2.7%]) (Figure 2). The mean length of stay for patients with leptospirosis-associated hospitalizations was longer than that for patients with nonleptospirosis infectious disease–associated hospitalizations (6.9 days [SE 0.4] vs. 5.6 days [SE 0.01]; p<0.001). The mean and median lengths of stay were not statistically significantly different by the age or sex of patients or by region.

For 1998–2009, the estimated hospital charges for leptospirosis-associated hospitalizations totaled US $76,013,667 (SE US $7,908,317). The mean charge for leptospirosis-associated hospitalizations was US $39,181 (SE US $3,493); this amount was significantly higher than the mean charge for nonleptospirosis infectious disease–associated hospitalizations (US $26,871 [SE US $198], p<0.001). The mean leptospirosis-associated hospitalization charges did not differ significantly between male patients (US $39,427 [SE US $4,231]) and female patients (US $38,605 [SE US $6,119]) or by age group (<20 years of age, US $40,659 [SE US $8,545]; 20–59 years of age, US $33,606 [SE US $3,351]; >60 years of age, US $53,533 [SE US $10,566]).

Leptospirosis-specific diagnoses were listed on the hospital records as follows (the percentage of records listing each diagnosis is shown in parentheses): unspecified leptospirosis (73% [SE 2.5%]); leptospirosis icterohemorrhagica (17% [SE 1.9%]); leptospirosis meningitis (6% [SE 1.3%]); and other specific *Leptospira* spp. infections (4% [SE 1.0%]). The most common diagnoses listed for leptospirosis-associated hospitalizations were volume depletion (23.8% [SE 2.1%]); thrombocytopenia, unspecified (18.7% [SE 2.0%]); acute kidney failure, unspecified (18.3% [SE 2.0%]); and fever and other physiologic disturbances of temperature regulation (13.1% [SE 1.7%]) (Table 3). Frequently performed procedures included spinal tap (20.5% [SE 2.1%]), venous catheterization (10.2% [SE 1.4%]), hemodialysis (7.4% [SE 1.3%]), and transfusion of packed cells (6.2% [SE 1.3%]) (Table 4).

**Table 3:** Selected diagnoses listed on leptospirosis-associated hospitalization discharge records, United States, 1998–2009*

Diagnosis†	ICD-9-CM code	No. (SE) discharge records	% (SE) discharge records
Volume depletion	276.5	475 (53)	23.8 (2.1)
Thrombocytopenia, unspecified	287.5	373 (50)	18.7 (2.0)
Acute kidney failure, unspecified‡	584.9	365 (48)	18.3 (2.0)
Fever and other physiologic disturbances of temperature regulation	780.6	261 (36)	13.1 (1.7)
Hyposmolality and/or hyponatremia	276.1	249 (37)	12.5 (1.6)
Hypopotassemia	276.8	203 (34)	10.2 (1.5)
Acute and subacute necrosis of liver‡	570	148 (28)	7.4 (1.4)
Jaundice, unspecified, not of newborn‡	782.4	124 (25)	6.2 (1.2)
Atrial fibrillation†	427.31	99 (24)	5.0 (1.1)
Acute respiratory failure‡	518.81	93 (21)	4.7 (1.1)

* ICD-9-CM, International Classification of Diseases, 9th revision, Clinical Modification (*17*). †Discharge records may contain >1 listed diagnosis. ‡Diagnoses commonly associated with leptospirosis.

**Table 4:** Most frequent procedures listed on leptospirosis-associated hospitalization discharge records, United States, 1998–2009*

Procedure†	ICD-9-CM code	No. (SE) discharge records	% (SE) discharge records
Spinal tap	03.31	408 (46)	20.5 (2.1)
Venous catheterization, not elsewhere classified	38.93	203 (32)	10.2 (1.4)
Hemodialysis	39.95	147 (27)	7.4 (1.3)
Transfusion of packed cells	99.04	124 (26)	6.2 (1.3)
Venous catheterization for renal dialysis	38.95	107 (23)	5.4 (1.2)

*ICD-9-CM, International Classification of Diseases, 9th revision, Clinical Modification (*17*). †Discharge records may contain >1 listed procedure.

## Discussion

The current incidence of leptospirosis in the United States is unknown because national surveillance of the disease ceased after 1994 (*10*). Reports of reemergence and increased incidence of leptospirosis in US states and globally (*1*,*5*,*11*,*12*), expanded number of risk groups (*7*,*8*), and a higher than expected death rate among reported case-patients in Puerto Rico (*13*) are raising concern that human leptospirosis infections may be on the rise in the United States. Thus, existing data must be used to estimate the number of cases nationwide. NIS is an available dataset that can be used to estimate the number of leptospirosis case-patients requiring hospitalization, evaluate trends of leptospirosis-associated hospitalizations, and compare parameters of leptospirosis-associated hospitalizations with those of nonleptospirosis infectious disease–associated hospitalizations.

The findings from our study indicate that the number of symptomatic patients with leptospirosis requiring hospitalization may be low in the United States. In addition, the findings do not indicate an increase in leptospirosis-associated hospitalizations over the study period, 1998–2009. However, the average annual leptospirosis-associated hospitalization rate of 0.6 hospitalizations/1,000,000 population likely represents only a proportion of all clinically diagnosed leptospirosis cases during 1998–2009, and the rate represents a much smaller proportion of all *Leptospira* spp. infections (*2*,*3*,*12*). Several studies have found that 70%–90% of patients with reported leptospirosis cases are hospitalized (*5*,*12*,*25*); however, an active surveillance study identified 5 times more leptospirosis cases than had been identified through passive surveillance, of which only 30% of the actively identified patients were hospitalized (*26*). Two studies of active case-finding following common-source outbreaks in the United States reported that 6% and 32% of the patients, respectively, were hospitalized (*8*,*9*). Although not directly comparable, the US leptospirosis incidence rate for 1994 (calculated from data in the Nationally Notifiable Diseases Surveillance System, http://wwwn.cdc.gov/nndss/) was 0.2 hospitalizations/1,000,000 population (*27*). The differences between the percentage of hospitalized patients identified from passive and active surveillance and between the leptospirosis-associated hospitalization rate and the 1994 leptospirosis incidence rate could indicate underrecognition of cases and underreporting of cases to the Nationally Notifiable Diseases Surveillance System.

Male patients were more likely than female patients to have a leptospirosis-associated hospitalization. The difference in disease occurrence between sexes has been established in the literature (*2*,*3*). Although the cause for this difference is not clear, it has often been ascribed to higher rates of exposure to *Leptospira* spp. among the male population (*2*,*3*); this higher exposure is reflected in labor statistics and in the demographics of recreational activities associated with leptospirosis outbreaks (*8*,*9*,*28*). A few studies have demonstrated increased hospitalization rates, disease severity, and leptospiremia among male patients, which may indicate greater susceptibility for severe disease in male patients (*25*,*29*). More research is needed to determine the reason for this disparity; however, it is likely multifactorial.

We found that persons >20 years of age were more likely than younger persons to have a leptospirosis-associated hospitalization. A lower incidence of infection in children has been widely reported (*6*,*12*,*25*,*26*,*30*,*31*); the difference is likely due to increased environmental exposure to the bacteria among adults (*3*,*4*).

Because the incubation period for leptospirosis is 1–2 weeks (range 2–30 days), the month of hospital admission for infected persons closely approximates the month of exposure to the pathogen (*2*). The distribution of hospitalizations by admission month in our study reflects the seasonality of leptospirosis infections (*2*–*4*). The predominance of leptospirosis cases in summer and fall has been linked to increased environmental exposure to the bacteria through contaminated water and soil during warm months (*2*,*4*) and through flooding events associated with hurricanes (*6*,*9*).

In our study, the median length of hospital stay for patients with leptospirosis-associated hospitalizations was 4.1 days; other studies have reported median lengths of stay of 5–10 days (range 1–46 days) (*5*,*26*). The higher hospital charges and longer lengths of stay for patients with leptospirosis-associated hospitalizations, compared with those for nonleptospirosis infectious disease–associated hospitalizations, likely result from the need for intensive care, supportive therapies, and invasive procedures that may be associated with the more severe form of leptospirosis. Support from an intensive care unit was required for 33%–64% of leptospirosis patients (*5*,*32*). In the presence of renal dysfunction and failure, which have been reported in 26%–47% of leptospirosis patients (*25*,*31*–*34*), fluid replacement therapy and dialysis are indicated to improve clinical outcome (*2*,*3*). The presence of hemorrhagic conditions, including hematuria, hematemesis, and hemoptysis, ranges from 9.1% to 81.5% in patients hospitalized for leptospirosis (*30*,*31*,*35*), and these conditions often require blood transfusions (*2*). These therapies and procedures increase the cost of patient care (*3*6), and such increases may be reflected in our study findings. Improved awareness among clinicians of the clinical signs and symptoms of leptospirosis may lead to earlier diagnosis and treatment of the disease, which may reduce disease severity and, thus, hospitalization charges (*36*).

In NIS, aggregate demographic data for hospitalizations with an ICD-9-CM code of 100 (leptospirosis) are similar to data reported in the literature for patients with leptospirosis, but the leptospirosis-specific diagnoses are lower than expected. The NIS data are likely a valid representation of leptospirosis patients, although they may overrepresent the number of patients hospitalized after the initial febrile phase has ended. However, the use of the NIS dataset has limitations. Data from >44 US states are included in the NIS each year (*14*); the incidence of leptospirosis may or may not be higher in the states not included in the annual sample. This limitation is especially pertinent to US territories, where leptospirosis is often an endemic disease (*6*,*37*). For these reasons, the overall rate of leptospirosis-associated hospitalization may have been underestimated. However, in 1998, the annual NIS sampling frame comprised 67% of all US hospitalizations, but by 2009, 95% of all hospitalizations were included. Also, there is potential for misdiagnosis or for miscoding on hospitalization records. The diagnoses (ICD-9-CM codes) are physician-based; neither laboratory confirmation of the diagnosis nor the reason for the hospitalization is included in the hospital discharge records.

Leptospirosis outbreaks have occurred in temperate and tropical areas of the United States, typically following flooding events (*8*,*9*,*11*,*12*). As flooding events occur, infections may go unrecognized, particularly when other concurrent febrile illness outbreaks are occurring (*6*,*12*,*13*). The reinstatement of leptospirosis as a nationally notifiable condition in the United States has enabled the establishment of leptospirosis surveillance and the collection of case data (*10*). These data will be used to calculate the national incidence of reported leptospirosis cases in the United States, clarify the current epidemiology of the disease, and possibly assess the benefit of earlier diagnosis and treatment on patient outcomes. In addition, changes in health outcomes may be reflected in future analyses of hospitalization data. Educating clinicians on the clinical signs and symptoms of leptospirosis and the importance of case reporting is needed; it may reduce possible underrecognition and underreporting of the disease.
